# JAK2 as Predictor of Therapeutic Response in Patients with Chronic Myeloid Leukemia Treated with Imatinib

**DOI:** 10.1155/2024/2906566

**Published:** 2024-04-12

**Authors:** Indra Wijaya, Muhammad H. Bashari, Lelani Reniarti, Anita Rahmawati, Rully M. A. Roesli

**Affiliations:** ^1^Division of Hematology and Medical Oncology, Department of Internal Medicine, Faculty of Medicine, Hasan Sadikin General Hospital, Universitas Padjadjaran, Bandung, Indonesia; ^2^Department of Biomedical Sciences, Faculty of Medicine, Universitas Padjadjaran, Bandung, Indonesia; ^3^Department of Child Health, Faculty of Medicine, Hasan Sadikin General Hospital, Universitas Padjadjaran, Bandung, Indonesia; ^4^Division of Nephrology and Hypertension, Department of Internal Medicine, Faculty of Medicine, Hasan Sadikin General Hospital, Universitas Padjadjaran, Bandung, Indonesia

## Abstract

**Background:**

Chronic myeloid leukemia (CML) or chronic granulocytic leukemia is a myeloproliferative neoplasm indicated by the presence of the Philadelphia (Ph+) chromosome. First-line tyrosine kinase inhibitor, imatinib, is the gold standard for treatment. However, there has been known unresponsiveness to treatment, especially due to the involvement of other genes, such as the Janus kinase 2 (JAK2) gene. This study aimed to evaluate the relationships between JAK2 levels and complete hematological response (CHR), as well as early molecular response (EMR) after 3 months of imatinib treatment in patients with chronic phase CML.

**Methods:**

Patients with Ph+ CML in the chronic phase (*n* = 40; mean age, 40 ± 11 years) were recruited to complete assessments consisting of clinical examination and blood test, including evaluation of complete blood counts and the JAK2 levels, at baseline and following 3 months of therapy with imatinib (at an oral dose of 400 mg per day). Subjects were divided into two groups according to the presence of CHR and EMR.

**Results:**

JAK2 gene levels, phosphorylated, and total JAK2 proteins at baseline were significantly lower in the group with the presence of CHR and EMR. In addition, baseline JAK2 levels, including JAK2 gene expression, phosphorylated, and total JAK2 proteins, were negatively correlated with the presence of CHR and EMR.

**Conclusions:**

Based on these findings, JAK2 levels may be a potential indicator for evaluating treatment response on imatinib due to its role in the pathophysiology of CML.

## 1. Introduction

Chronic myeloid leukemia (CML) or chronic granulocytic leukemia is a myeloproliferative neoplasm indicated by the presence of the Philadelphia (Ph+) chromosome and its oncogenic gene, BCR-ABL. The Ph+ is a result of a genetic mutation, known as a reciprocal translocation of the c-ABL segment of chromosome 9 and breakpoint cluster region (BCR) of chromosome 22 (*t* (9; 22) (q34; q11)) [1–3]. CML accounts for 20%–30% of leukemia cases in adults and ranges between 55 and 60 years. Its incidence is approximately 10–15 cases per 1,000,000 adults per year [1].

The BCR-ABL gene induces genetic instability by stimulating tyrosine kinase enzyme, leading to proliferation, transformation, apoptosis suppression, and disruption of adhesion of hematopoietic cells, especially myeloid series in bone marrow stroma [2, 4]. The BCR-ABL gene expression is an essential factor in evaluating the growth and survival of leukemic cells. Additionally, it correlates with CML severity clinically [5, 6].

Treatment of CML remains challenging. First-line tyrosine kinase inhibitors, such as imatinib, are the gold standard for CML therapy [1, 7–9]. Imatinib induces remissions in 97%, and the overall survival reaches 93% [10–12]. However, 33% of cases treated with imatinib were not responsive to therapy. Potential factors affecting unresponsiveness to treatment is the involvement of other genes, such as the Janus kinase 2 (JAK2) gene, as previously reported [13–17].

The JAK2 gene is included in the Janus kinase family, which consists of four genes, JAK1, JAK2, JAK3, and JAK4. In normal conditions, the JAK2 gene has an important role in the regulation of the hematopoietic process through a signaling pathway involving cytoplasmic tyrosine kinase, which furthermore activates signal transduction and activator of transcription (STAT), some of the ras-mitogen-activated protein kinase (MAPK), and phosphoinositide 3–kinase (PI3K) pathways. JAK2 regulates the maturation, proliferation, apoptosis, and differentiation of myeloid cells [18–21]. Studies have shown that JAK2 gene dysregulation is related to several hematologic malignancies, in terms of initiation or progressivity [22, 23]. In addition, in the previous investigations, the role of disruption of JAK2 gene expression has been established in other diseases, such as polycythemia vera, essential thrombosis, and primary myelofibrosis [18, 24, 25].

It has been reported that JAK2 may have a potential role in the pathophysiological mechanism of CML. In the in vitro and in vivo studies, Xie et al. [26] found that there was a bonding between JAK2 and ABL, which resulted in JAK2 phosphorylation [26]. JAK2 also increased Myc expression, which leads to the antiapoptotic capability of CML cells [27]. Additionally, Gallipoli et al. [28] found that JAK2 inhibitors affected JAK-STAT pathway activity in vitro by inducing apoptosis of CML progenitor cells.

Given the efficacy of imatinib in reducing the burden of CML, treatment monitoring is necessary. According to European LeukemiaNet (ELN), National Comprehensive Cancer Network (NCCN), and European Society for Medical Oncology (ESMO) guidelines for the treatment of CML, evaluation of therapeutic responses of imatinib is recommended by using complete hematological response (CHR) and early molecular response (EMR) after 3 months of treatment. On the other hand, regarding the potential role of JAK2 in the pathophysiology of CML and treatment efficacy, it may be valuable to assess the levels of JAK2 gene expression or JAK2 protein. A previous case-control study by Gorre et al. [16] showed that an increase in JAK2 gene expression occurred at the diagnosis of CML and during treatment, but no significant relationship was found between JAK2 gene expression and molecular response [16]. However, they did not measure total JAK2 protein and phosphorylated JAK2 levels. Therefore, the actual activity of JAK2 could not be determined. Additionally, the measurement of JAK2 gene expression was conducted once. Little is known regarding the JAK2 gene or JAK2 protein expression in CML patients following imatinib therapy. Hence, this study's objectives were to evaluate the relationships between JAK2 gene expression, total JAK2 protein, phosphorylated JAK2 levels, and CHR, as well as EMR before and after 3 months of imatinib treatment in patients with chronic phase CML. The advantages of these examinations were to clarify the role of JAK2 at all levels, from gene expression to protein and phosphorylated levels, and to evaluate the effects of imatinib therapy on the JAK2 level changes. Furthermore, the assessment of these therapeutic response parameters was very important in determining whether the treatment was optimal or not.

## 2. Materials and Methods

### 2.1. Subjects

This study was a prospective cohort study. Only patients with a diagnosis of Ph+ CML in the chronic phase were consecutively recruited during their admission in outpatient or inpatient care at the Division of Hematology and Medical Oncology, Department of Internal Medicine, Hasan Sadikin General Hospital, Bandung. The inclusion criterion was patients who were newly diagnosed with Ph+ CML in the chronic phase and would be treated with imatinib (Gleevec, Novartis Pharma, New Jersey, USA). Diagnosis of CML was established based on the clinical history, hematological examination, and molecular diagnosis confirmed by using real-time reverse transcriptase quantitative polymerase chain reaction quantitative (RT Q-PCR) for the BCR-ABL fusion gene. The chronic phase was described by an increase in granulocytes with the presence of less than 10% blasts in the peripheral blood smear [12, 29]. Imatinib was given at an oral dose of 400 mg per day. It was fully covered by government health insurance. Patients who were on acute infection or received steroids or other immunosuppressant agents were excluded.

### 2.2. Variables and Measurements

Data from recruited patients was recorded at two time points, at baseline (before imatinib treatment) and follow-up (after 3 months of imatinib treatment). We acquired demographic variables consisting of age and sex, as well as clinical data, including spleen size. Three prognostic methods, Sokal et al. [30], the European Treatment and Outcome Study for CML (EUTOS) [31], and Hasford et al. [32] risk scores at baseline were examined according to patients' baseline characteristics, including age, spleen size, and differential cell count. A Sokal et al. [30] score of <0.8 indicates low risk, a score of 0.8–1.2 intermediate risk, and a score of >1.2 high risk [11, 30]. A Hasford et al. [32] score of ≤780 indicates low risk, 780–1,480 intermediate risk, and >1,480 high risk. Regarding the EUTOS score, Hasford et al. [31] previously defined the prognostic score of ≤87 as low risk and >87 as high risk.

The patient's blood samples were collected through venipuncture into ethylene diamine tetraacetic acid (EDTA) vacutainer by a trained laboratory technician. The blood samples were used for the evaluation of complete blood counts, BCR-ABL transcripts, and JAK2 levels. An automated hematology analyzer (Sysmex XT2100i) was used to measure parameters for hematological response, including hemoglobin, white blood cells, peripheral blood basophils, eosinophils, platelet count, and peripheral blood blasts. Additionally, BCR-ABL transcripts were evaluated by AccuPower® BCR–ABL RT Q-PCR kit from Bioneer. This resulted in the BCR-ABL ratio based on an international scale.

We assessed the JAK2 levels in patients before imatinib treatment and after 3 months of imatinib treatment. The assessments were performed in the Division of Molecular Biology, Department of Clinical Pathology, Hasan Sadikin General Hospital, Bandung. The parameters included the levels of JAK2 gene expression, phosphorylated, and total JAK2 proteins. Investigation of mRNA transcript of the JAK2 gene was performed by RT Q-PCR, while phosphorylated JAK2 and total JAK2 proteins were measured by enzyme-linked immunosorbent assay (ELISA) method.

Expression of the JAK2 gene was analyzed using the determination of Taqman chemistry. A blood sample was used for DNA isolation by nonenzymatic/RNA salting out by using the TRIzol method. Conversion of isolated RNA into cDNA conducted through one-step reverse transcription (Invitrogen cDNA conversion kit). RT Q-PCR was performed on an ABI Step One Real-Time PCR machine. JAK2 gene assay ID was Hs01078120_m1 with the size of the amplicon of 119 bp.

The concentration of phosphorylated JAK2 and total JAK2 proteins were quantified using commercial ELISA assay kits from Invitrogen for human JAK2 (Phospho) (pY1007/pY1008) and Human JAK2 kit E–EL–H2239 (Elabscience Biotechnology, US), respectively. The cell lysate for the assessment was from a blood sample after being washed with phosphate-buffered saline (PBS) solution and then added to the lysis buffer solution. The assay was conducted according to the manufacturer's instructions. Calibration curves were constructed by plotting the absorbance values at 450 nm vs. the phosphorylated JAK2 or total JAK2 concentrations of the calibrators, and concentrations of samples were determined by using the calibration curve.

Imatinib was administrated in a standard dose of 400 mg/day. After 3 months of imatinib treatment, CHR and EMR were assessed to evaluate treatment response. Treatment response after the first 3 months is valuable in the management of CML because it predicts the success of treatment in the following months [33–35] and is also useful for defining clinical strategies [36]. In this study, patients were divided into two groups according to the presence of CHR and EMR, respectively. The positive presence of CHR was defined by leukocytes ≤10,000/mL, platelets ≤450,000/mL, with a normal differential blood count, basophils <5%, no immature cells (myelocytes, promyelocytes, or blasts) in peripheral blood, and absence of spleen enlargement [2, 8, 37]. The positive presence of EMR was defined as a BCR-ABL transcript level ratio ≤10% on an international scale (glyceraldehyde 3-phosphate dehydrogenase, GADPH, in this study) at 3 months [8, 16, 36, 37].

### 2.3. Statistical Analysis

We assessed the Gaussian distribution of the data using a Shapiro–Wilk test. Results were presented as means ± standard deviations (normally distributed data) or median and range (non-normally distributed data) for continuous variables, as well as number (percentage) for categorical variables. Differences between the two groups were analyzed using Student's *t*-test (Mann–Whitney *U*-test, if appropriate) and *Chi*-square test (Fisher's exact test, if appropriate). Correlation coefficients (*r*) for the JAK2 levels and presence of CHR, as well as EMR, were obtained using point-biserial Pearson product–moment correlations or Spearman's rho correlation test as appropriate. Evaluation of the JAK2 levels at baseline (before imatinib treatment) performance at predicting treatment response after 3 months of imatinib treatment was carried out using a comparison of the presence of CHR and EMR to the baseline JAK2 levels. The performance metrics of the baseline JAK2 levels were assessed for sensitivity, specificity, positive predictive value, negative predictive value, likelihood ratios, and the area under the receiver operating characteristic (ROC) curve (AUC) when compared with the presence of CHR and EMR. A *p* value < 0.05 was considered statistically significant and a two-sided test was used. All statistical analyses were performed using SPSS 24.0 (SPSS, Chicago, IL, USA).

## 3. Results

### 3.1. Patient Characteristics

Of the 43 attending the study, three patients were not eligible because of loss of follow-up after 3 months of imatinib treatment. The baseline characteristics of the 40 patients are demonstrated in Table 1. The total group comprised 24 males and 16 females with a mean age of 40 ± 11 years. Most patients had splenomegaly (spleen size of 8 ± 3 cm), light anemia (9.5 ± 2.6 g/dL), and leukocytosis (median 111,585, range 20,390–498,470). We used three prognostic systems to estimate treatment outcomes from baseline characteristics. The majority of patients, 42.5% and 95.0%, had low risk, according to Sokal et al. [30] and EUTOS risk scores, respectively. On the other hand, based on the Hasford risk score, patients were mostly included in intermediate-risk groups (37.5%).

### 3.2. Complete Hematological Response (CHR) Evaluation

Twenty-seven patients (67.5%) achieved CHR within 3 months. The comparisons of baseline characteristics and JAK2 levels stratified by the presence of CHR are summarized in Table 2. There were no differences in terms of demographic and laboratory parameters in the two groups. Sokal et al. [30], Hasford et al. [31], and EUTOS score risk groups did not influence hematological response in this study. All patients with the presence of CHR had low EUTOS risk at diagnosis. Regarding the levels of JAK2 before imatinib treatment, JAK2 gene expressions in the group with the presence of CHR were significantly lower than those of the group with the absence of CHR (14.90 (0.18–60.53) vs. 44.01 (9.78–100.40), *p* < 0.001). In line with this, the levels of phosphorylated and total JAK2 proteins at baseline were significantly lower in the group with CHR (0.48 (0.03–0.09) vs. 0.80 (0.24–1.34), *p*=0.005 and 69.92 (11.82–535.27) vs. 427.56 (45.98–2,291.00), *p*=0.002, respectively). After 3 months of imatinib use, only phosphorylated JAK2 protein showed a significantly lower value in the group with the presence of CHR (0.48 (0.01–1.10) vs. 0.93 (0.24–13.95), *p*=0.009).

In terms of correlation analysis, as shown in Table 3, JAK2 levels, including baseline JAK2 gene expression, phosphorylated, and total JAK2 proteins were negatively correlated with the presence of CHR (*r* = −0.333, *p*=0.018; *r* = −0.448, *p*=0.002; and *r* = −0.477, *p*=0.001, respectively). In addition, there was a significantly strong and negative correlation between JAK2 gene levels after 3 months of imatinib therapy and the presence of CHR (*r* = −0.605, *p* < 0.001) in patients with chronic phase CML. In protein levels, only phosphorylated JAK2 protein showed a significantly negative correlation with the presence of CHR after 3 months of imatinib use (*r* = −0.476, *p*=0.001).

Concerning predicting the presence of CHR after 3 months of imatinib therapy, baseline phosphorylated JAK2 protein levels had a sensitivity of 0.59 and a specificity of 0.85 when the cutoff of ≤0.58 was used (Table 4). The AUC was 0.78. When the assessment was based on the baseline total JAK2 protein with the cutoff of ≤370.79 pg/mL, the sensitivity and specificity were 0.93 and 0.54, respectively, with an AUC of 0.80.

### 3.3. Early Molecular Response (EMR) Evaluation

Twenty-four patients (60.0%) achieved EMR after 3 months of imatinib therapy.  Table 5 lists the comparison results of patient characteristics and JAK2 levels between the two groups; presence and absence of EMR. Regarding patient characteristics, only platelet count was significantly lower in the group with the presence of EMR (*p*=0.024). Sokal, Hasford, and EUTOS score risk groups did not significantly affect molecular response in this study. However, all patients with the presence of EMR had low EUTOS risk scores at baseline. In two comparisons, patients with EMR had significantly lower JAK2 gene expression (14.35 (0.18–35.13) vs. 43.86 (9.78–100.40), *p* < 0.001), phosphorylated JAK2 protein (0.40 (0.03–0.90) vs. 0.80 (0.24–1.34), *p*=0.005), and total JAK2 protein (61.07 (11.82–370. 79) vs. 451.66 (45.98–2,291.00), *p*=0.002) at baseline compared with those without EMR. Regarding JAK2 levels after 3 months of imatinib therapy, only phosphorylated JAK2 protein was significantly lower in the group with the presence of EMR (0.28 (0.01–1.10) vs. 0.80 (0.24–13.95), *p*=0.001).

Table 3 shows the correlation between JAK2 levels (before and after imatinib treatment) and the presence of EMR. There were significant negative correlations between JAK2 levels before imatinib treatment with the presence of EMR (*r* = −0.271, *p*=0.045 for JAK2 gene; *r* = −0.543, *p* < 0.001 for phosphorylated JAK2; and *r* = −0.520, *p* < 0.001 for total JAK2 protein). In addition, there was a significantly strong negative correlation between JAK2 gene expression after 3 months of imatinib treatment and EMR (*r* = −0.654, *p* < 0.001). Phosphorylated JAK2 protein after 3 months of imatinib treatment had a moderate negative correlation with EMR (*r* = 0.421, *p*=0.003) in patients with chronic phase CML.

To estimate the presence of EMR after 3 months of imatinib treatment, baseline phosphorylated JAK2 protein had a sensitivity of 0.75 and a specificity of 0.81 when the cutoff of ≤0.70 was used (Table 4). The AUC was 0.82. When the assessment was according to the baseline total JAK2 protein with a cutoff of ≤187.48, the sensitivity and specificity were 0.83 and 0.75, respectively, with an AUC of 0.87.

## 4. Discussion

In this study, JAK2 gene expression and phosphorylated JAK2 level were lower in groups with CHR or EMR at baseline, as well as 3 months after imatinib treatment. Moreover, inverse correlations among levels of JAK2 gene expression, phosphorylated, and total JAK2 proteins at baseline with the presence of hematological response or molecular responses were demonstrated in our study. JAK2 gene is included in the JAK family that has a role in the regulation of the hematopoietic process by encoding cytoplasmic tyrosine kinase enzyme and activates signal transduction, mainly STAT pathway and some of Ras–MAPK and PI3K pathway [18–21]. A previous study by Gorre et al. [16] reported that there was no significant relationship between JAK2 gene expression and hematological or molecular responses, but an increase in JAK2 gene expression occurred when the diagnosis of CML was made and during treatment. The results of the previous study differed presumably because of the smaller number of subjects. In addition, only JAK2 gene expression was assessed without measuring total JAK2 protein and phosphorylated JAK2 levels in the previous study. Therefore, the actual activity of JAK2 could not be determined.

CML is a clonal myeloproliferative neoplasm that progresses over three phases: chronic phase, accelerated phase, and blast crises [2, 3, 36]. Most CML is diagnosed in the chronic phase [8]. BCR-ABL gene fusion, with constitutive tyrosine kinase activity, has been found in almost 90% of CML cases and is consistently related to cell morphology, clinical manifestation, and laboratory parameters [38]. Therefore, tyrosine kinase inhibitors, such as imatinib, have been a gold standard of CML treatment [1, 7–9, 39]. The efficacy of imatinib in CML management reached an estimation of 81% of disease-free survival and 93% of overall survival [3, 12, 40].

Patients in this study were diagnosed as having Ph+ CML in the chronic phase and received imatinib with a dose of 400 mg per day. Most patients in this study were male. It is consistent with previous reports regarding gender differences in the epidemiology of CML [41]. The mean age at diagnosis in this study was 40 years, ranging from 39 to 55. These ages were younger compared with other studies that showed the mean age of patients diagnosed with CML of 55–60 years [1, 3]. Age at diagnosis is related to the CML prognosis. Aging-related comorbidity, treatment-related complication, or age-related contraindication of bone marrow transplant may explain the worse prognosis by older age. Administration of tyrosine kinase inhibitors has beneficial effects on life span [42, 43].

Determination of risk score using the Sokal, Hasford, or EUTOS scoring system before initiation of tyrosine kinase inhibitor therapy is recommended for patients with chronic phase CML [8]. Additionally, in CML treatment, it is essential to evaluate the treatment response after the first 3 months. It can predict the treatment failure or success in the following months [33–35] and is valuable in defining clinical strategies [36]. In this study, we used CHR and EMR to evaluate the treatment response after 3 months of treatment. The cytogenetic response was not performed due to the limitation of facilities and financial support. In the present study, patients mostly showed the presence of CHR and EMR (67.5% and 60.0%, respectively). It suggests that imatinib is effective at the hematological and molecular level of response in the chronic phase of CML. However, based on previous studies, treatment with imatinib with a standard dose of 400 mg affects higher CHR from 88% to 98% [44, 45]. Differences in CHR or EMR may be explained by patients' adherence to treatment, which is dependent on age, sex, education level, disease condition, and tolerance of side effects [46].

Regarding the subjects' characteristics, our study found no differences in the characteristics of patients with and without the presence of CHR or EMR. However, other studies showed different results, such as differences in white blood cells and hemoglobin at baseline before reaching CHR [47]. Although prognostic score systems in CML, including Sokal et al. [30], Hasford et al. [31], and EUTOS were developed to predict major molecular response and complete cytogenetic response, several studies also investigated their relationships with CHR [48, 49]. Patients with low Sokal et al. [30] and Hasford et al. [31] scores were more likely to achieve CHR [49]. In the present study, the EUTOS score showed a trend of risk stratification in achieving CHR or EMR, although not significant.

Previous work has demonstrated 33% of cases with unresponsiveness to imatinib therapy [50]. JAK2 activity is one of the potential contributors that has been investigated previously [13–17]. This study found that JAK2 levels, including baseline JAK2 gene expression, phosphorylated, and total JAK2 proteins, were negatively correlated with the presence of CHR and EMR after 3 months of treatment with imatinib. This is in line with the study by Gorre et al. [16] which found that JAK2 gene expression is elevated in CML patients at diagnosis and during the course of treatment. Therefore, JAK2 gene expression analysis at the time of diagnosis might help in predicting imatinib response.

There is emerging evidence that JAK2 may play a role in the pathogenesis of CML. Higher levels of the JAK2 expression and activity were related to increased proliferation activity of hematopoietic cells, including leukemic cells. Leukemic cells have resembled receptors of proliferation signals. Moreover, overactivity of the JAK2 receptor affects uncontrolled hematopoietic cell proliferation and is in line with abnormality of clinical manifestation (splenomegaly) and hematological parameters, such as leukocytosis and thrombocytosis. Several studies have shown the hyperactivity of JAK2 and the role of JAK2 in CML progressivity [4, 16, 51]. In CML patients, the transformation of BCR-ABL induces the activation of JAK2 through phosphorylation. Xie et al. [26, 27] have reported that JAK2 and ABL bonding could elicit JAK2 phosphorylation, which was not affected by BCR-ABL tyrosine kinase inhibitors, such as imatinib. JAK2 also increased Myc expression related to the antiapoptotic activity of CML cells [26, 27].

When baseline phosphorylated JAK2 protein levels ≤0.58 as a cutoff to predict CHR after 3 months of imatinib therapy, high specificity, but low sensitivity was found in this study. On the other hand, based on the baseline total JAK2 protein, with the cutoff of ≤370.79 pg/mL, high sensitivity, but low specificity was found. Similarly, baseline phosphorylated and total JAK2 proteins seem to be less robust in predicting EMR after 3 months of imatinib treatment. The quantification of the best cutoff of JAK2 levels to predict treatment responses needs a prospective study with a greater number of subjects and varied conditions. Assessment of these therapeutic response parameters is very important in determining whether the treatment was optimal or not. This will determine whether imatinib will be continued or should be replaced with a next-generation tyrosine kinase inhibitor drug to achieve a better prognosis.

There were some limitations in this study. JAK2 activation could be elicited by stimulation to cytokine or hematopoietic receptors, including growth hormones, prolactin, thrombopoietin, leptin, cardiotrophin-1, oncostatin-M, granulocyte-macrophage colony-stimulating factor, interleukin (mainly 3 and 6), and interferón-gamma mediators [21, 52]. However, we have excluded patients with the inflammatory condition or acute infection, as well as subjects with clinical signs of infection and under steroid use. On the other hand, compliance information was not systematically collected in our study. We did not analyze imatinib resistance due to BCR-ABL gene mutation or overexpression in the group without CHR and EMR. Those factors may affect the therapeutic responses. In addition, this was a single-center study with a limited number of subjects and a follow-up period. Further longitudinal study with a greater number of subjects is necessary. Regardless of the limitations, our study complemented previous reports related to the role of JAK2 in CML.

## 5. Conclusions

In conclusion, our results indicated that the levels of JAK2 gene expression, phosphorylated, and total JAK2 proteins at the time of diagnosis correlated with the presence of CHR and EMR in patients with chronic phase CML treated with 3 months of imatinib. Therefore, assessment of the levels of JAK2 gene expression, phosphorylated, and total JAK2 proteins at the time of diagnosis could be considered in predicting therapeutic responses among patients with chronic phase CML treated with imatinib. Based on these findings, JAK2 levels may be a potential indicator for evaluating treatment response on imatinib due to its role in the pathophysiology of CML.

## Figures and Tables

**Table 1:** Patient baseline characteristics.

Variable	*n* = 40
Age, years (mean ± SD)	40 ± 11
Sex, *n* (%)	
Male	24 (60)
Female	16 (40)
Spleen size, cm (mean ± SD)	8 ± 3
Laboratory parameters, median (range)	
Hemoglobin (g/dL)	9.5 ± 2.6
White cell count (/*μ*L)	111,585 (20,390–498,470)
Peripheral blood basophils (%)	1 (0–8)
Eosinophils (%)	1 (0–5)
Platelet count (x10^3^/*μ*L)	294 (31–1,123)
Peripheral blood blasts (%)	8 (2–20)
Sokal risk groups, *n* (%)	
Low (<0.8)	17 (42.5)
Intermediate (0.8–1.2)	7 (17.5)
High (>1.2)	16 (40.0)
Hasford risk groups, *n* (%)	
Low (≤780)	12 (30.0)
Intermediate (781–1,480)	15 (37.5)
High (>1,480)	13 (32.5)
EUTOS risk groups, *n* (%)	
Low risk (≤87)	38 (95.0)
High risk (>87)	2 (5.0)

**Table 2:** Patient characteristics and JAK2 levels stratified by the presence of complete hematological response groups.

Variable	Complete hematological response		
	Present (*n* = 27)	Absent (*n* = 13)	*p*-Value
Age, years (mean ± SD)	42 ± 12	37 ± 8	0.261
Sex, *n* (%)	—	—	0.895
Male	16 (59.3)	8 (61.5)	—
Female	11 (40.7)	5 (38.5)	—
Spleen size, cm (mean ± SD)	9 ± 3	7 ± 2	0.167
Laboratory parameters, mean ± SD or median (range)			
Hemoglobin (g/dL)	9.8 ± 2.5	8.7 ± 2.6	0.208
White blood cells (/*μ*L)	92,000 (20,390–498,000)	142,250 (33,000–344,130)	0.231
Peripheral blood basophils (%)	1 (0–8)	1 (0–7)	0.467
Eosinophils (%)	1 (0–5)	1 (0–4)	0.285
Platelet count (x10^3^/*μ*L)	223 (31–1,123)	445 (49–960)	0.153
Peripheral blood blasts (%)	8 (4–19)	8 (2–20)	0.685
Sokal risk groups, *n* (%)	—	—	0.941
Low (<0.8)	11 (40.7)	6 (46.2)	—
Intermediate (0.8–1.2)	5 (18.6)	2 (15.4)	—
High (>1.2)	11 (40.7)	5 (38.4)	—
Hasford risk groups, n (%)	—	—	0.701
Low (≤780)	7 (26.0)	5 (38.4)	—
Intermediate (781–1,480)	11 (40.7)	4 (30.8)	—
High (>1,480)	9 (33.3)	4 (30.8)	—
EUTOS risk groups, *n* (%)	—	—	0.100
Low risk (≤87)	27 (100.0)	11 (84.6)	—
High risk (>87)	0 (0.0)	2 (15.4)	—
JAK2 levels before imatinib treatment, median (range)			
JAK2 gene (fold changes)	14.90 (0.18–60.53)	44.01 (9.78–100.40)	<0.001
Phosphorylated JAK2 (U/mL)	0.48 (0.03–0.90)	0.80 (0.24–1.34)	0.005
Total JAK2 (pg/mL)	69.92 (11.82–535.27)	427.56 (45.98–2,291.00)	0.002
JAK2 levels after 3 months of imatinib treatment, median (range)			
JAK2 gene (fold changes)	0.70 (0.00–8.80)	1.20 (0.10–39.10)	0.271
Phosphorylated JAK2 (U/mL)	0.48 (0.01–1.10)	0.93 (0.24–13.95)	0.009
Total JAK2 (pg/mL)	22.69 (2.92–282.50)	32.16 (5.49–461.89)	0.613

**Table 3:** Correlation coefficients for JAK2 levels and the presence of a complete hematological response, as well as early molecular response.

Variable	Complete hematological response		Early molecular response	
	*r*	*p*-Value	*r*	*p*-Value
JAK2 levels before imatinib treatment				
JAK2 gene	−0.333	0.018	−0.271	0.045
Phosphorylated JAK2	−0.448	0.002	−0.543	<0.001
Total JAK2	−0.477	0.001	−0.520	<0.001
JAK2 levels after 3 months of imatinib treatment				
JAK2 gene	−0.605	<0.001	−0.654	<0.001
Phosphorylated JAK2	−0.476	0.001	−0.421	0.003
Total JAK2	−0.141	0.192	−0.157	0.166

**Table 4:** Sensitivity, specificity, positive predictive value (PPV), negative predictive value (NPV), and area under the receiver operating characteristic (ROC) curve (AUC) of the presence of complete hematological response and early molecular response using cutoff values of baseline JAK2 levels (before imatinib treatment).

Variable	Cutoff	Sensitivity	Specificity	PPV	NPV	AUC (95% CI)	*p*-Value
Complete hematological response							
JAK2 gene	≤0.90	0.63	0.62	0.77	0.44	0.61 (0.44–0.76)	0.30
Phosphorylated JAK2	≤0.58	0.59	0.85	0.89	0.50	0.78 (0.62–0.89)	<0.001
Total JAK2	≤370.79	0.93	0.54	0.81	0.78	0.80 (0.64–0.91)	<0.001
Early molecular response							
JAK2 gene	≤0.80	0.63	0.63	0.71	0.53	0.58 (0.41–0.73)	0.45
Phosphorylated JAK2	≤0.70	0.75	0.81	0.85	0.69	0.82 (0.67–0.92)	<0.001
Total JAK2	≤187.48	0.83	0.75	0.84	0.75	0.87 (0.72–0.95)	<0.001

**Table 5:** Patient characteristics and JAK2 levels stratified by the presence of early molecular response groups.

Variable	Early molecular response		
	Present (*n* = 24)	Absent (*n* = 16)	*p*-Value
Age, years (mean ± SD)	40 ± 12	40 ± 10	0.986
Sex, *n* (%)	—	—	0.792
Male	14 (58.3)	10 (62.5)	—
Female	10 (41.7)	6 (37.5)	—
Spleen size, cm (mean ± SD)	9 ± 3	7 ± 2	0.104
Laboratory parameters, mean ± SD, or median (range)			
Hemoglobin (g/dL)	9.4 ± 2.1	9.5 ± 3.2	0.892
White blood cells (/*μ*L)	100,150 (20,390–498,470)	131,475 (33,000–344,130)	0.456
Peripheral blood basophils (%)	1 (0–8)	1 (0–7)	0.836
Eosinophils (%)	1 (0–5)	1 (0–5)	0.614
Platelet count (x10^3^/*μ*L)	199 (31–803)	462 (49–1,123)	0.024
Peripheral blood blasts (%)	8 (4–19)	9 (2–20)	0.709
Sokal risk groups, *n* (%)	—	—	0.936
Low (<0.8)	10 (41.7)	7 (43.8)	—
Intermediate (0.8–1.2)	4 (16.6)	3 (18.8)	—
High (>1.2)	10 (41.7)	6 (37.4)	—
Hasford risk groups, *n* (%)	—	—	0.987
Low (≤780)	7 (29.2)	5 (31.3)	—
Intermediate (781–1,480)	9 (37.5)	6 (37.4)	—
High (>1,480)	8 (33.3)	5 (31.3)	—
EUTOS risk groups, *n* (%)	—	—	0.154
Low risk (≤87)	24 (100.0)	14 (87.5)	—
High risk (>87)	0 (0.0)	2 (12.5)	—
JAK2 levels before imatinib treatment, median (range)			
JAK2 gene (fold changes)	14.35 (0.18–35.13)	43.86 (9.78–100.40)	<0.001
Phosphorylated JAK2 (U/mL)	0.40 (0.03–0.90)	0.80 (0.24–1.34)	0.005
Total JAK2 (pg/mL)	61.07 (11.82–370.79)	451.66 (45.98–2,291.00)	0.002
JAK2 levels after 3 months of imatinib treatment, median (range)			
JAK2 gene (fold changes)	0.70 (0.00–8.00)	1.20 (0.00–39.00)	0.422
Phosphorylated JAK2 (U/mL)	0.28 (0.01–1.10)	0.80 (0.24–13.95)	0.001
Total JAK2 (pg/mL)	23.17 (2.92–282.50)	26.64 (5.49–461.89)	0.804

## Data Availability

The data used to support the findings of this study are available from the corresponding author upon request.
